# Prostate cancer disease recurrence after radical prostatectomy is associated with HLA type and local cytomegalovirus immunity

**DOI:** 10.1002/1878-0261.13273

**Published:** 2022-08-31

**Authors:** Johanna Classon, Margherita Zamboni, Camilla Engblom, Kanar Alkass, Giulia Mantovani, Christian Pou, Dieudonné Nkulikiyimfura, Petter Brodin, Henrik Druid, Jeff Mold, Jonas Frisén

**Affiliations:** ^1^ Department of Cell and Molecular Biology Karolinska Institutet Stockholm Sweden; ^2^ Department of Pathology and Oncology Karolinska Institutet Stockholm Sweden; ^3^ Science for Life Laboratory, Department of Women's and Children's Health Karolinska Institutet Stockholm Sweden

**Keywords:** antiviral protection, cytomegalovirus (CMV), HLA‐alleles, prognosis, prostate cancer, T‐cells

## Abstract

Prostate cancer is a heterogeneous disease with a need for new prognostic biomarkers. Human leukocyte antigen (HLA) genes are highly polymorphic genes central to antigen presentation to T‐cells. Two alleles, HLA‐A*02:01 and HLA‐A*24:02, have been associated with prognosis in patients diagnosed with *de novo* metastatic prostate cancer. We leveraged the next‐generation sequenced cohorts CPC‐GENE and TCGA‐PRAD to examine HLA alleles, antiviral T‐cell receptors and prostate cancer disease recurrence after prostatectomy. Carrying HLA‐A*02:01 (111/229; 48% of patients) was independently associated with disease recurrence in patients with low‐intermediate risk prostate cancer. HLA‐A*11 (carried by 42/441; 10% of patients) was independently associated with rapid disease recurrence in patients with high‐risk prostate cancer. Moreover, HLA‐A*02:01 carriers in which anti‐cytomegalovirus T‐cell receptors (CMV‐TCR) were identified in tumors (13/144; 10% of all patients in the cohort) had a higher risk of disease recurrence than CMV‐TCR‐negative patients. These findings suggest that HLA‐type and CMV immunity may be valuable biomarkers for prostate cancer progression.

## Graphical abstract

In this study, we find that variants of genes that are essential for the immune system (HLA‐A*02:01 and HLA‐A*11) are associated with prostate cancer progression after radical prostatectomy. Local detection of immunity to cytomegalovirus, but not EBV or Influenza A, was associated with particularly low disease recurrence‐free survival, suggesting that HLA‐type and viral immunity could predict cancer progression.

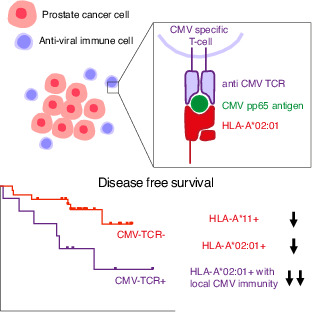

**Abbreviations**
AU: arbitrary units
AUC: area under the curve
BCR: B cell receptor
EBV: Epstein–Barr virus
EBV‐TCR: EBV‐BMLF1‐GLC
CI: confidence interval
CMV: human cytomegalovirus
CMV‐TCR: CMV‐pp65‐NLV
CPM: counts per million
Flu: influenza A
Flu‐TCR: Flu‐M‐GIL TCR
GIL: GILGFVFTL
GLC: GLCTLVAML
HLA: human leukocyte antigen
HR: hazard ratio
MHC: major histocompatibility complex
NLV: NLVPMVATV
PSA: prostate specific antigen
ROC: receiver operating characteristic
RPKM: reads per kilobase per million
s‐PSA: serum‐prostate specific antigen
TCR: T‐cell receptor
WGS: whole genome sequencing

## 1 Introduction

Prostate cancer is one of the most common cancers in men, with clinical features ranging from long‐term indolence to rapid disease recurrence and death. Clinical and histological tumor features at diagnosis can only moderately stratify the risk of disease progression [1]. Besides rare germline mutations in DNA repair genes and prostate‐specific antigen (PSA), few prognostic biomarkers are known [2].

Prostate cancer is classically considered an immunologically cold tumor but is nevertheless infiltrated by many immune cell types, including T‐cells [3]. The major histocompatibility complex, the most polymorphic locus of the human genome, contains human leukocyte antigen (HLA) genes that are fundamental for antigen presentation to T‐cells. This polymorphism determines a person's possible repertoire of differentiated T‐cells. The resulting repertoire is for example defined by foreign antigens from encountered viruses. In a small European cohort with *de novo* metastatic prostate cancer (*n* = 56), HLA‐A*02:01 and HLA‐A*24:02 have been suggested to have prognostic implications [4], but beyond that, little is known about the role of HLA‐type and antiviral immunity in prostate cancer. Human cytomegalovirus (CMV), a common herpes virus, is a master modulator of the immune system [5]. Cytomegalovirus has been implicated in tumor biology [6, 7], but its role in prostate cancer is unclear.

## 2 Materials and methods

### 2.1 Ethical approval

The study was conducted in accordance with the principles of the Declaration of Helsinki. Analysis of primary prostate cancer next‐generation sequencing datasets CPC‐GENE and TCGA‐PRAD was permitted by the Swedish Ethical Review Authority (2019–03086, 2020–01374). Access to CPC‐GENE data was granted through the International Cancer Genome Consortium and access to TCGA‐PRAD data were granted through dbGaP. The experiments were undertaken with the understanding and written consent of each subject. Ethical permit for the study of human samples was granted by the Regional ethics committee in Stockholm (2010/313‐31/3) and the Swedish Ethical Review Authority (2019‐03086). Oral informed consent for donation, as per Swedish law, was given by relatives prior to inclusion in the study. The informed consent was documented in writing.

### 2.2 Next‐generation sequencing prostate cancer cohorts

Clinical data and per genome alterations in tumors for patients in CPC‐GENE were compiled from published information [8, 9, 10]. Prostate cancer patients were recruited for the study between 2011 and 2016. In CPC‐GENE, disease recurrence (*n* = 53) was defined as biochemical recurrence (*n* = 52) or development of metastatic disease (*n* = 13). One patient developed metastatic disease in the absence of biochemical recurrence (a patient that was subject to whole genome sequencing). In CPC‐GENE, biochemical recurrence was defined as two consecutive measurements of serum‐PSA (s‐PSA) greater than 0.2 ng·mL^−1^ with time to biochemical recurrence being from prostatectomy to the first measured rise in s‐PSA [8] or as described in [11] if a patient was treated with salvage therapy. Mutation counts per tumor and frequencies of specific mutations in tumors of HLA‐A*02:01^+^ and HLA‐A*02:01^−^ patients in CPC‐GENE were downloaded from cBioPortal (cbioportal.org 20210902).

TCGA‐PRAD RNA‐sequencing cohort was partly published in [12] and generated by the TCGA Research Network: https://www.cancer.gov/tcga. Prostate cancer patients were diagnosed with cancer between 2000 and 2013. Clinical data for patients in TCGA‐PRAD was downloaded and compiled from the National cancer institute GDC Data Portal using the files ‘nationwidechildrens.org_clinical_follow_up_v1.0_prad’ and ‘nationwidechildrens.org_clinical_patient_prad’. These patients were excluded in TCGA‐PRAD: no sequence files were available for HLA‐typing, time to recurrence event was not documented, M1 disease at prostate cancer diagnosis, new tumor event was recorded as “new primary tumor, non‐prostatic” or the type of new tumor event was unavailable. In the TCGA‐PRAD cohort, disease recurrence was defined as the first documented signs of disease recurrence and could be biochemical evidence of disease (*n* = 79; one both biochemical recurrence and locoregional recurrence), locoregional recurrence (*n* = 4; one both biochemical recurrence and locoregional recurrence) or development of metastatic disease (*n* = 3).

For CPC‐GENE and TCGA‐PRAD cohorts, all available patients matching the inclusion and exclusion criteria stated above were analyzed. No power calculations were made to decide on sample size. For broad analysis of HLA allele groups and prostate cancer, disease‐free survival allele groups with an allele frequency of above 5% were analyzed, with the assumption that allele groups with lower frequency would include too few patients.

### 2.3 HLA‐typing of next‐generation sequencing data

To infer HLA allele genotypes from RNA‐sequencing data, fastq files were aligned to the human genome (GRCh38, release 99) using STAR (v2.5.3a). ArcasHLA [13], version 0.2.0, was then used in combination with IMGT/HLA database 3.39.0 to extract reads mapped to classical HLA genes (HLA‐A, HLA‐B, HLA‐C, HLA‐DPB1, HLA‐DRB1, HLA‐DQA1, HLA‐DQB1) and to perform HLA‐type inference. Default parameters were used for ‘arcasHLA extract’ and ‘arcasHLA genotype’ functions. Whole genome sequencing samples were mapped against the human genome (GRCh38) using Burrows‐Wheeler Alignment Tool (bwa mem, v0.7.8) with default settings. Picard SortSam (v2.10.3) was then used to sort reads by coordinate and Picard MarkDuplicates with optional ‘‐‐VALIDATION_STRINGENCY=SILENT’ tag was used to identify duplicate reads. Finally, HLA‐LA (v1.0.1, [14] was used with PRG_MHC_GRCh38_withIMGT graph to infer HLA allele genotypes.

### 2.4 Viral TCR identification and gene expression analysis in RNA‐sequenced tumors

mixcr [15], version 3.0.12, and vdjmatch, version 1.3.1, were used to infer T‐cell receptor (TCR) and B‐cell receptor (BCR) chain sequences and summary statistics in read pairs. A wider knowledge of epitope specificity exists for TCRs compared to BCRs, and we therefore focused on TCRs, in particular, TCRβ chains due to their high complexity. mixcr ‘analyze shotgun’ pipeline was carried out on paired‐end fastq files using ‘rna’ as ‘‐‐starting‐material’ and default parameters. The TCR clonotype table obtained from mixcr was subsequently used as input to vdjmatch to compare the clonotypes present in the sample against TCR sequences of known antigen specificity (VDJdb) [16, 17]. vdjmatch was run separately for TRB and TRA genes (e.g., −R TRB), and it was carried out once with default parameters and once after filtering for CMV‐specific epitopes (−‐filter=“__antigen.species__=~‘CMV’”).

### 2.5 Gene expression analysis in RNA‐sequenced tumors

RNA‐seq samples were mapped against a combined reference, for which human (GRCh38) and virus (NC_006273.2) genomes had been concatenated, using STAR (v2.5.3a) and the ‘‐‐ sjdbGTFtagExonParentTranscript Parent’ flag. A gene‐expression matrix was then created with the CPC‐GENE samples aligned to the combined reference using featureCounts (v2.0.0). Subsequently, data were log‐normalized and differential expression analysis was carried out using Seurat's (v3.2.2) FindMarkers function with the Wilcoxon Rank Sum test. Significantly differentially expressed genes were determined according to these thresholds: average log2 (Fold Change) < 1.0 or > 1.0, *P* < 0.05. Functional enrichment analyses were performed using g:Profiler (version e103_eg50_p15_68c0e33) with g:SCS multiple testing correction method [18]. Proportions of 22 immune cell types were determined by cibersortx using the CIBERSORTx website [19].

### 2.6 Statistical analyses

The following analyses were performed in graphpad prism 8 (GraphPad Software, San Diego, CA, USA). The difference in disease recurrence‐free survival between groups in univariate analyses were compared with Log‐rank (Mantel‐Cox) test and plotted as Kaplan–Meier survival curves or as hazard ratios with a 95% confidence interval. For screening associations between HLA groups (one‐field) and disease recurrence‐free survival in TCGA‐PRAD, Benjamini–Hochberg procedure with a false discovery rate of 5% resulted in only HLA‐A*11 being significantly associated with prognosis. In TCGA‐PRAD, the three patients who had metastatic disease at inclusion were excluded from the univariate analyses of disease recurrence‐free survival. Groups with continuous numerical values were compared by two‐tailed un‐paired Student's *t*‐test or two‐tailed Mann–Whitney test depending on data normality. Categorical data between two groups were compared with two‐sided Fisher's exact test. A correlation between total TCRβ, determined with MiXCR reads (CPM) and CD3e gene expression (RPKM) was examined by Spearman correlation. T‐cell marker expression and normalized reads were compared in three groups with the non‐parametric Kruskal–Wallis test. VirScores were compared between serum and prostate with linear regression.

Multivariate survival analyses (Cox proportional hazard models) of disease recurrence in HLA‐A*02:01^+^ compared to HLA‐A*02:01^−^ patients in CPC‐GENE and HLA‐A*11^+^ compared to HLA‐A*11^−^ patients in TCGA‐PRAD were performed in R using survfit and coxph functions from ‘survival’ package, version 3.2–11. In CPC‐GENE, pre‐treatment s‐PSA (< 10, > 10), pathological Gleason grade group (1–3, 4–5) and cT‐stage (T1, T2) were used in a Cox proportional hazard model. In TCGA‐PRAD, pre‐treatment s‐PSA (< 10, 10–20, > 20), clinical Gleason grade group (1–3, 4–5), pT‐stage (T1–T2, T3–T4) and N‐stage (0/1) were used in a Cox proportional hazard model in patients with no missing data.

Receiver operating characteristic (ROC) analyses were performed in spss (v28) (IBM, Armonk, NY, USA). Binary logistic regression was performed using clinical variables included in the Cox proportional hazard models. The resulting regression statistics for the models were used in ROC analyses for disease recurrence.

### 2.7 Human postmortem serum and prostate tissue

Blood and prostate tissue were obtained from postmortem donors undergoing autopsy at the Department of Forensic Medicine in Stockholm from 2015 to 2019. A slice of postmortem collected prostate was cut transversal, to ensure that the three anatomical regions of the prostate were included. One side of the prostate was frozen at −80 °C. Serum was separated from other blood products by centrifugation at 20 min, 2700 ***g*** at 4°. The presence of prostate cancer in donated prostates was determined by histological assessment by a trained pathologist, who also retrieved and extracted relevant medical information about the donors from autopsy reports and medical records.

### 2.8 Protein extraction and CMV IgG analysis of serum and prostate

For analysis of antiviral antibodies in the prostate, proteins were extracted from frozen tissue in RIPA buffer (R0278, Sigma–Aldrich, Saint Louis, MO, USA) containing a complete protease inhibitor (COEDTAF‐RO, Sigma–Aldrich). Tissues were either dissociated and homogenized manually with a syringe and lysed rocking at 4 °C at 1 h or homogenized using homogenizing beads (Precellys 2 mL tissue homogenizing mixing beads kit, 10409, Cayman Chemical, Ann Arbor, MI, USA) in a Precellys 24 homogenizer and incubated at 4 °C for 20 min. Tissue lysates were centrifuged and supernatants were recovered. Protein concentration was determined with Pierce BCA Protein Assay Kit (23225, ThermoFisher, Waltham, MA, USA) according to manufacturer's instructions. Total IgG levels in prostate and serum samples were measured by a human IgG ELISA kit (RAB0001, Sigma–Aldrich). Prostate protein lysates (100 μg) were assayed using the human anti‐cytomegalovirus IgG ELISA Kit (CMV; ab108639, Abcam, Cambridge, UK) according to manufacturer's instructions. Anti‐CMV IgG titer in serum was analyzed using CMV IgG, CMIA assay (Architect, Abbott, Chicago, IL, USA), performed at Karolinska University Hospital Clinical Microbiology Laboratory. The cut‐off value was 6.0 arbitrary units (AU) and measured values up to 250.

### 2.9 VirScan

VirScan is a high‐throughput method to comprehensively analyze antiviral antibodies using immunoprecipitation and massively parallel DNA sequencing [20]. The bacteriophage display library that included viruses known to have human tropism (in total 206 species in the library and > 1000 strains was shared by the Elledge laboratory (Department of Genetics, Harvard University Medical School, Boston, MA, USA) and propagated/expanded in the Brodin laboratory (Science for Life Laboratory, Karolinska Institutet, Stockholm, Sweden). The bacteriophage display library was designed to present 56‐amino‐acid‐long linear peptides that overlap by 28 amino acids. Prostate protein lysate and serum samples corresponding to 2 μg total IgG were used as input for VirScan analysis, performed as described in [21]. RIPA buffer was used as a negative control. In general, technical replicates of samples were incubated together with the bacteriophages followed by magnetic bead capture of IgG‐bacteriophage complexes. Immunoprecipitated bacteriophages were lysed and sequenced by Illumina sequencing at the National Genomics Infrastructure, SciLifeLab (HiSeq Rapid Mode single read 1 × 50 base pairs). Sequencing reads were processed as in Pou et al. [21] to produce a list of non‐enriched (0) and enriched (1) peptides. Peptide hits found in beads‐only negative control or RIPA buffer negative control were removed from the analysis. Cross‐reactive antibodies were excluded, as described in [21]. The resulting matrix with VirScores per viral species in each sample was used for further analysis and data visualization. Two prostate samples were run in replicates, and a mean value of their respective VirScores was used.

VirScore of 1 was defined as one non‐contiguous epitope hit. Total VirScore per virus reflects the number of epitopes recognized by antibodies in a sample. The possibility of detecting a larger number of epitope‐specific antibodies, and therefore getting a higher VirScore, is higher in large viruses (e.g., CMV and EBV) compared to smaller viruses (e.g., Enterovirus B) [20]. VirScan is prone to produce background/non‐specific VirScore hits [20]. We therefore only included prostate samples with VirScore > 5 for a virus in a heatmap. All prostate VirScore hits are shown in Table S6.

## 3 Results

### 3.1 Classical HLA genes are rarely mutated in primary prostate cancer

**Fig. 1:**
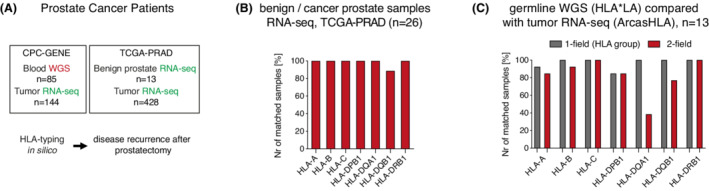
Classical HLA genes are rarely mutated in primary prostate cancer. (A) Illustration of study design and HLA‐typing. (B) Comparison of HLA‐typing of seven classical HLA genes (two‐field) between 26 pairs of RNA‐sequenced benign prostate and prostate cancer showed very high concordance. Samples were HLA‐typed with arcasHLA. (C) Comparison of HLA‐type in 13 pairs of blood whole genome sequencing and tumor RNA‐sequencing. HLA*LA was used for HLA‐type whole genome sequencing data and arcasHLA was used for HLA‐type RNA‐sequencing data. [Colour figure can be viewed at wileyonlinelibrary.com]

To examine if HLA‐A*02:01 and HLA‐A*24:02 are associated with prostate cancer progression after radical prostatectomy, we analyzed two prospective North American cohorts: one with mainly early‐stage (AJCC 8^th^ edition clinical stage I–II; 97%; CPC‐GENE, *n* = 229) and one with mainly later‐stage prostate cancer (AJCC 8^th^ edition clinical stage III–IV; 68%, TCGA‐PRAD, *n* = 441; *n* = 345 with data that allowed clinical staging; Fig. 1A, Table S1). Most disease recurrence events after surgery were only evident by PSA recurrence and just a few patients developed locoregional recurrence or distant metastases during the limited follow‐up time of median 6.8 years in CPC‐GENE and median 2.1 years in TCGA‐PRAD (Table S1). Patients' HLA alleles were determined with recently developed bioinformatic HLA‐typing tools using RNA‐ and/or whole genome‐sequencing data from blood (CPC‐GENE: *n* = 85), benign prostate tissue (TCGA‐PRAD: *n* = 13) or primary tumors (CPC‐GENE: *n* = 144, TCGA‐PRAD: *n* = 428; Fig. 1A). Comparing HLA‐type in 26 matched RNA‐sequenced benign prostate tissue and primary prostate tumors, we found that germline and tumor HLA‐types (two‐field) were identical in all HLA‐genes examined except for a few mismatches in HLA‐DQB1 (Fig. 1B). HLA‐typing in matched blood by whole genome sequencing and primary tumors by RNA‐sequencing, HLA groups (one‐field) were identical except for HLA‐DPB1 in two patients and HLA‐A in one patient (Fig. 1C and Fig. S1A). These results support previous studies reporting low HLA mutation frequency in primary prostate cancer [22, 23].

### 3.2 Carriers of particular HLA‐types have poor disease‐free survival after radical prostatectomy

**Fig. 2:**
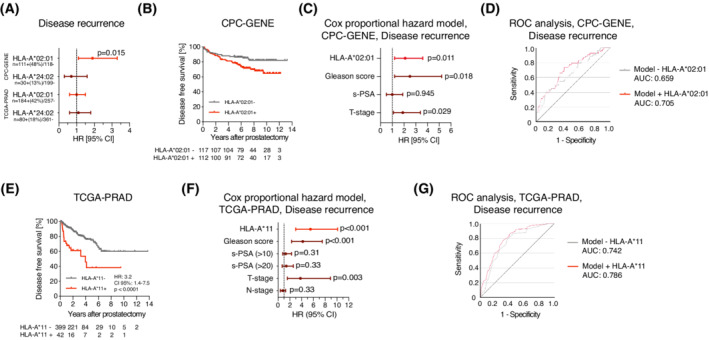
Carriers of particular HLA‐types have poor disease‐free survival after radical prostatectomy. (A) Forest plot showing prostate cancer disease‐free survival after prostatectomy in HLA‐A*02:01^+^ compared to HLA‐A*02:01^−^ patients and HLA‐A*24:02^+^ compared to HLA‐A*24:02^−^ patients using a log‐rank test. (B) Kaplan–Meier curve showing disease recurrence‐free survival of HLA‐A*02:01^−^ patients (*n* = 118) and HLA‐A*02:01^+^ patients (*n* = 111) in CPC‐GENE. Statistical measures are shown in (A). Patients at risk are shown underneath the graph. (C) Forest plot of Cox proportional hazard model including the variables HLA‐A*02:01 (0/1), Gleason score (grade group 1–3/4–5), pre‐treatment s‐PSA (< 10/> 10), T‐stage (cT1/cT2). *n* = 229. (D) ROC curve showing predictability of disease recurrence in CPC‐GENE in binary logistic regression models with Gleason score, pre‐treatment s‐PSA, T‐stage and HLA‐A*02:01. AUC = Area under the curve. (E) Kaplan–Meier curve showing disease recurrence‐free survival of HLA‐A*11^−^ patients (*n* = 399) and HLA‐A*11^+^ patients (*n* = 42) in TCGA‐PRAD, statistically compared using a log‐rank test. Patients at risk are shown underneath the graph. (F) Forest plot showing Cox proportional hazard model in TCGA‐PRAD with co‐variates HLA‐A*11 (0/1), Gleason score (grade group 1–3/4–5), pre‐treatment s‐PSA (< 10/10–20/> 20), T‐stage (pT1‐2/pT3‐4) and N‐stage (0/1). This analysis was only performed in patients with no missing data (*n* = 336). (G) ROC curve showing predictability of disease recurrence in TCGA‐PRAD in binary logistic regression models with Gleason score, pre‐treatment s‐PSA, T‐stage, N‐stage and HLA‐A*11. AUC = Area under the curve. In (A), (C) and (F), the dotted line delineates HR 1.0. and error bars are CI 95% = 95% confidence interval. HR = Hazard ratio. [Colour figure can be viewed at wileyonlinelibrary.com]

In CPC‐GENE, HLA‐A*02:01 carriers (111/229; 48%) had a higher risk of disease recurrence than HLA‐A*02:01 negative patients in a univariate (*P* = 0.015, HR: 1.9 CI 95%: 1.1–3.3; Fig. 2A,B) and a multivariate analysis adjusted for Gleason score, clinical T‐stage and pre‐treatment s‐PSA (Fig. 2C; C‐index without HLA: 0.64, C‐index 0.67 with HLA). In a ROC analysis, the addition of HLA‐A*02:01 to a model with clinical variables increased its ability to predict disease recurrence (AUC increased from 0.659 to 0.705; Fig. 2D). Furthermore, HLA‐A*02:01 was not associated with Gleason score, T‐stage or pre‐treatment s‐PSA (Table S2). The few HLA‐A*02:01 homozygous patients (*n* = 16) did not have a higher risk of disease recurrence than HLA‐A*02:01 heterozygous patients (Fig. S2A). HLA‐A*24:02 (carried by 13/229; 13%) was not associated with disease recurrence (Fig. 2A). Examining all HLA allele groups with an allele frequency of > 5%, no other allele group than HLA‐A*02 was associated with disease recurrence in CPC‐GENE (Fig. S2B, Table S3). The TCGA‐PRAD study design allowed discrimination of patients developing rapid disease recurrence from those who did not. Notably, neither HLA‐A*02:01 nor HLA‐A*24:02 were associated with recurrence in TCGA‐PRAD (Fig. 2A), in which the HLA allele group HLA‐A*11 (carried by 42/441; 9.5% of patients) instead was an independent factor associated with a high risk of rapid disease recurrence (Fig. 2E,F; C‐index without HLA: 0.70, C‐index: 0.75 with HLA, S2C, Tables S4 and S5). In a ROC analysis, the addition of HLA‐A*11 to a model with clinical variables increased its ability to correctly identify disease recurrence (AUC increased from 0.742 to 0.786; Fig. 2G). Treatment with adjuvant therapies (radiation or hormone therapy) after surgery did not alter the risk of rapid disease recurrence in HLA‐A*02:01 carriers, but too few patients were HLA‐A*11 carriers for this analysis (Fig. S2D,E). Perhaps the short follow‐up time (median 2.1 years) and high frequency of positive surgical margins in TCGA‐PRAD prohibited validation of HLA‐A*02:01 as a biomarker of prostate cancer progression. The potential clinical use of HLA‐type as prognostic biomarkers will be important to explore further.

### 3.3 HLA‐A*02:01 is not associated with a particular prostate cancer mutation profile

**Fig. 3:**
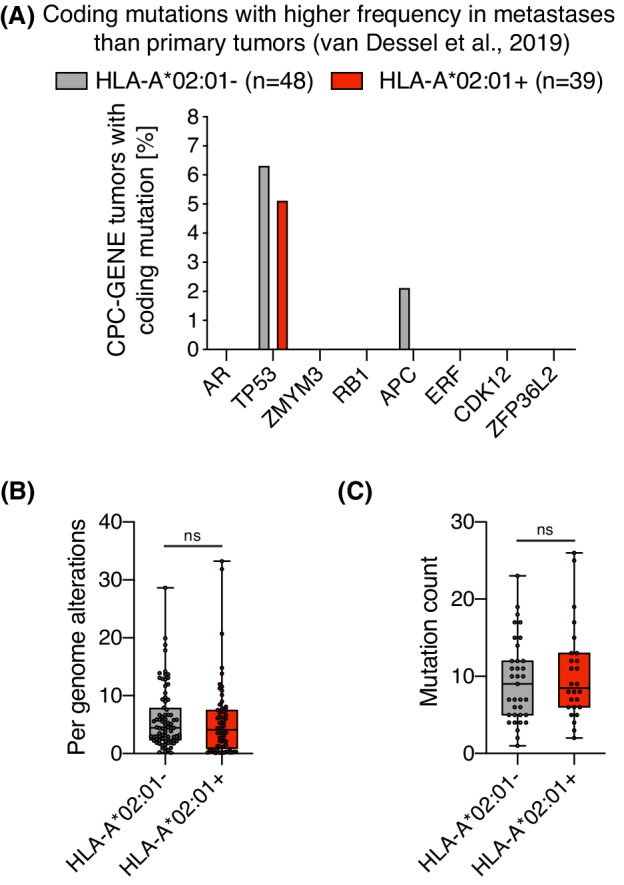
HLA‐A*02:01 is not associated with a particular prostate cancer mutation profile. (A) Frequencies of coding mutations, as described in [25], were determined in cbioportal and evaluated for HLA‐A*02:01^+^ (*n* = 39) and HLA‐A*02:01^−^ (*n* = 48) patients. (B) Per genome alterations in tumors, as described in [8], were compared between HLA‐A*02:01^−^ (*n* = 80) and HLA‐A*02:01^+^ (*n* = 64) patients using a Mann–Whitney test. (C) Mutation count per tumors, as documented in cBioPortal, compared between HLA‐A*02:01^−^ (*n* = 35) and HLA‐A*02:01^+^ (*n* = 26) patients using a Mann–Whitney test. Ns = non significant. The box plot shows 25 percentile, median, 75 percentile and error bar shows the minimum and maximum values. All values are shown in graphs. [Colour figure can be viewed at wileyonlinelibrary.com]

We then examined if we could discern why HLA‐A*02:01 may promote prostate cancer progression. HLA genes are classically viewed as restrictors and permitters of mutations in cancer, due to their ability to be hidden or visible for T‐cells as they are presented in the context of MHC complexes. Certain HLA alleles are associated with the mutational landscape in cancer [24], but among coding mutations associated with progression (mutations in AR, TP53, ZMYM3, RB1, APC, ERF, CDK12, ZFP36L2) [25], none were more common in primary tumors of HLA‐A*02:01 carriers (Fig. 3A). Furthermore, HLA‐A*02:01 carriers in CPC‐GENE did not have a higher tumor mutation burden compared to non‐carriers (Fig. 3B,C), which implies that HLA‐A*02:01 does not permit a large number of prostate cancer mutations to clonally expand through lack of immune recognition. Taken together, the risk of recurring disease in HLA‐A*02:01 carriers may not be mediated by the MHC complex role as a gatekeeper of mutation presentation that dictates selective immune recognition. The suppressive immune environment in prostate cancer may limit the role of HLA as a determinant of selective pressure.

### 3.4 Antiviral adaptive immunity is present in benign and malignant prostate

**Fig. 4:**
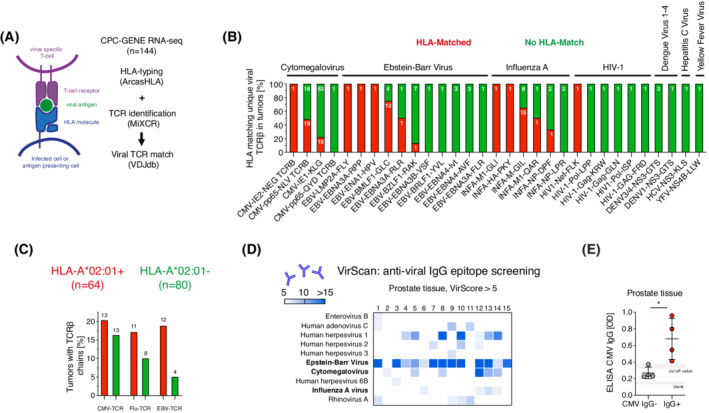
Antiviral immunity is detected in postmortem prostates and prostate cancer. (A) Illustration and description of the method in which antiviral T‐cell receptors were detected in RNA‐sequencing data from the CPC‐GENE cohort. VDJdb is VDJ database. (B) Percentage of tumors with HLA matched and HLA unmatched TCRβ detected by VDJdb. Each individual epitope is presented. In each bar, the number in white shows the total number of unique TCRβs found for the particular epitope. *N* = 144. (C) Bar graph showing that both HLA matched (HLA‐A*02:01^+^; *n* = 64) and HLA unmatched (HLA‐A*02:01^−^; *n* = 80) CMV‐TCR, Flu‐TCR and EBV‐TCR can be found analyzing tumor TCRβs. (D) VirScan (see Materials and methods) revealed 10 viruses with > 5 VirScore in prostate tissues from postmortem donors (*n* = 15) with varying prevalence. Samples 1–15 are shown individually on the heat map. The heat map is colored white (≤ 5 VirScore) and light blue to dark blue (> 5 VirScore) depending on the total VirScore per sample. Epstein–Barr Virus (EBV), Cytomegalovirus (CMV) and Influenza A (Flu) are highlighted. (E) ELISA for CMV IgG on prostate protein lysates from CMV IgG^+^ (*n* = 4) and CMV IgG^−^ (*n* = 4) men (CMIA CMV IgG assay in serum was used to define CMV IgG positivity) shows that all prostates from serum CMV IgG^+^ men, but no prostates from serum CMV IgG^−^ men were positive for CMV IgG. Cut‐off values and blank values are shown in gray. A Mann–Whitney test was performed to compare the difference in optical density. Line shows the mean and error bars show the standard deviation. * is *P* < 0.05. [Colour figure can be viewed at wileyonlinelibrary.com]

Since HLA‐A*02:01 did not promote prostate cancer progression via a cancer cell intrinsic mode, we turned to cancer cell extrinsic functions. Since HLA‐type defines a restricted T‐cell repertoire, we examined the role of HLA‐dependent adaptive immunity to pathogens in prostate cancer progression. Memory T‐cells can respond to reinfections or reactivation events of chronic viruses and can be found throughout the body in exposed individuals [26, 27]. It is unclear if an adaptive antiviral immune response is present in the prostate gland. In CPC‐GENE tumors (RNA‐seq, *n* = 144), the number of unique TCRβ chains, identified with MiXCR, ranged from 1 to 271 (median 31; Fig. 4A and Fig. S3A,B), supported by varying numbers of reads (Fig. S3B,C). As expected, TCRβ counts normalized to read depth positively correlated with expression levels of *CD3e*, a proxy marker for the number of T‐cells (Fig. S3D). TCRβ sequences were compared to the VDJ database that includes TCRβ specific for 12 viruses (Fig. 4A and Fig. S3E), resulting in one detected HLA matched HIV‐1 TCRβ and multiple HLA matched TCRβs for human cytomegalovirus (CMV), Epstein–Barr virus (EBV) and Influenza A (Flu; Fig. 4B). TCRβ with known specificity against the HLA‐A*02:01 restricted antigens CMV‐pp65‐NLVPMVATV(NLV) (referred to as CMV‐TCR), EBV‐BMLF1‐GLCTLVAML(GLC) (referred to as EBV‐TCR) and Flu‐M‐GILGFVFTL(GIL) (referred to as Flu‐TCR) were detected in tumors of 13 (20%), 12 (19%), and 11 (17%) of 64 HLA‐A*02:01^+^ patients, respectively (Fig. 4C).

Antiviral TCR identification via RNA‐sequencing is not yet a routine method. Therefore, we wished to validate that an antiviral immune response can indeed exist in prostate tissue using other techniques. Although B‐cell receptors can be detected with RNA‐sequencing and MiXCR, their epitope specificity is mainly unknown. To this end, we examined B‐cell mediated antiviral immunity using a high‐throughput antiviral IgG antibody screening method, called VirScan [20], in prostate tissue from 15 subjects collected postmortem, of whom one had been diagnosed with prostate cancer. We indeed detected B‐cell mediated immunity to CMV, Influenza A and EBV among others in the antiviral IgG antibody screen (Fig. 4D and Fig. S3F,G, Table S6). The presence of CMV IgG antibodies in the prostate corresponded to their presence in serum (Fig. S3F,G) and the presence of CMV IgG antibodies in prostates of CMV IgG^+^ donors was validated with ELISA (Fig. 4E).

### 3.5 Local CMV immunity is associated with poor disease‐free survival after radical prostatectomy

**Fig. 5:**
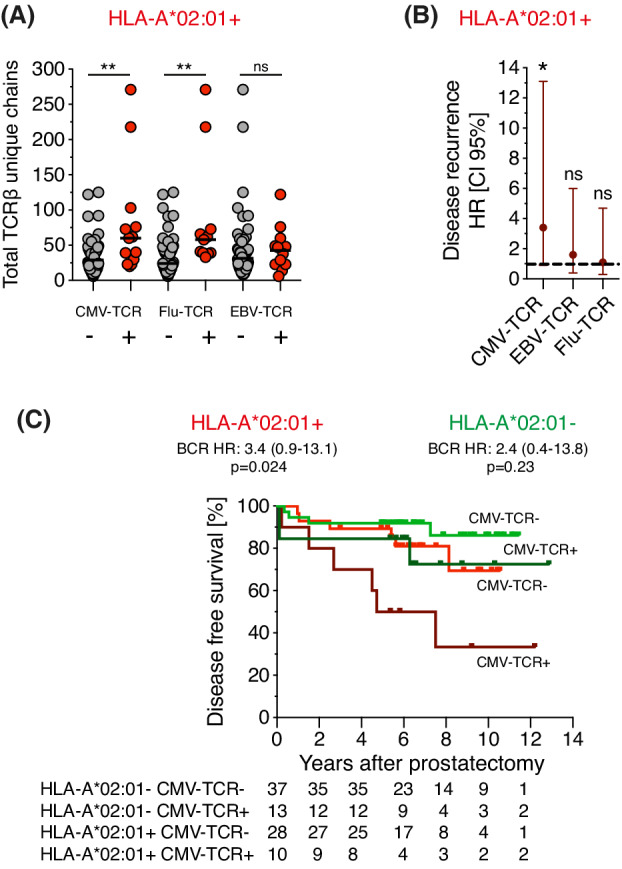
Local CMV immunity is associated with poor disease‐free survival after radical prostatectomy. (A) In HLA‐A*02:01^+^ patients (*n* = 64), the number of unique TCRβ chains per sample was higher in CMV‐TCR^+^ and Flu‐TCR^+^ compared to CMV‐TCR^−^ and Flu‐TCR^−^ samples respectively. Mann–Whitney tests were used to assess differences. Each dot represents one sample, the line shows median. ** is *P* < 0.01, ns is non significant. (B) Forest plot showing survival analyses of disease‐free survival with log‐rank tests analyzing HLA‐A*02:01^+^ patients with or without CMV‐TCR (> 23 unique TCRβ; negative: *n* = 28; positive: *n* = 10), EBV‐TCR (> 15 unique TCRβ; negative: *n* = 41; positive: *n* = 10) and Flu‐TCR (> 38 unique TCRβ; negative: *n* = 19; positive: *n* = 9), respectively. Patients with detected TCRβs above‐set thresholds were analyzed. HR = Hazard ratio. Error bar is CI 95% = 95% confidence interval. The dotted line delineates HR 1.0. * is *P* < 0.05, ns is non significant. (C) Kaplan–Meier curve showing disease‐free survival in HLA‐A*02:01 matched and unmatched CMV‐TCR^−^ and CMV‐TCR^+^ patients with > 23 unique detected TCRβs. Survival analyses were performed using log‐rank tests. Bright green is HLA‐A*02:01^−^ CMV‐TCR^−^; dark green is HLA‐A*02:01^−^ CMV‐TCR^+^; red is HLA‐A*02:01^+^ CMV‐TCR^−^; dark red/brown is HLA‐A*02:01^+^ CMV‐TCR^+^. Patients at risk are shown underneath the graph. [Colour figure can be viewed at wileyonlinelibrary.com]

HLA matched CMV‐TCR^+^ and Flu‐TCR^+^ tumors contained a higher number of total detected TCRβs than CMV‐TCR^−^ and Flu‐TCR^−^ tumors, respectively (Fig. 5A), indicating that discovery of antiviral TCRs is biased towards tumors with medium to high numbers of TCRβs. To reduce the risk of falsely allocating patients with a low number of TCRβs as antivirus TCR^−^, we examined patients with the top 80% of HLA‐matched antiviral TCRs and their TCRβ abundance matched controls in further analyses. The presence of CMV‐TCR was associated with recurrence in HLA‐A*02:01 carriers (*P* = 0.02, HR: 3.4, CI 95% 0.9–13.1, *n* = 38, Fig. 5B,C). In contrast, the presence of EBV‐TCR (*P* = 0.39) or Flu‐TCR (*P* = 0.86) was not associated with recurrence (Fig. 5B and Fig. S4A,B). HLA‐matched CMV‐TCR positive and negative prostate cancer patients had a similar distribution of clinical features at diagnosis (Table S7).

### 3.6 The association between CMV‐TCR and disease recurrence is independent of high tumor T‐cell infiltration

**Fig. 6:**
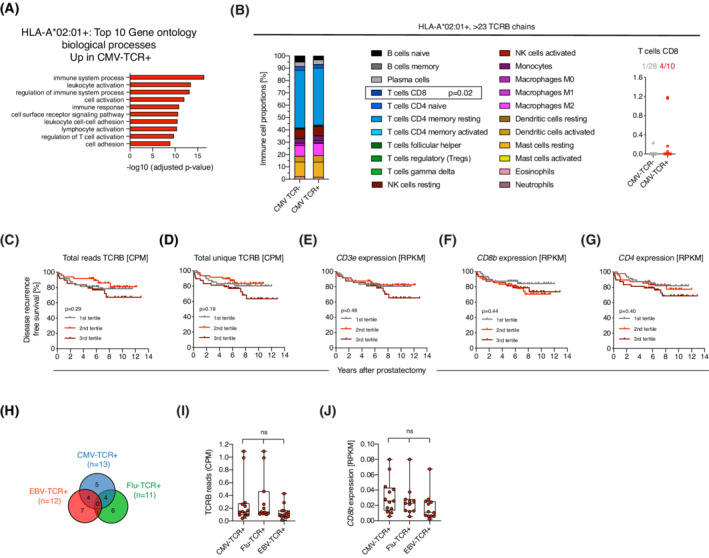
The association between CMV‐TCR and disease recurrence is independent of high tumor T‐cell infiltration. (A) Top 10 upregulated gene ontology biological processes in CMV‐TCR^+^ compared to CMV‐TCR^−^ tumors in HLA‐A*02:01^+^ prostate cancer samples with > 23 unique detected TCRβs. Bars show −log10 (adjusted *P*‐value) of statistical analysis in g:Profiler. (B) Profiling of immune cell proportions in prostate cancer RNA‐seq samples using cibersortx. CMV‐TCR^+^ (*n* = 28) and CMV‐TCR^−^ (*n* = 10) tumors in HLA‐A*02:01^+^ prostate cancer samples with > 23 unique detected TCRβs were compared and plotted as stacked bar plots. Percentage of immune cells were compared between the two groups by Mann–Whitney tests. CD8^+^ T‐cells were detected in low proportions by cibersortx in CMV‐TCR^−^ and CMV‐TCR^+^ prostate cancer samples. (C–G) Kaplan–Meier curves show analysis of T‐cell levels in tumors and disease‐free survival in the CPC‐GENE RNA‐sequencing cohort (*n* = 144). Total TCRβ reads and total unique TCRβ chains identified with MiXCR were normalized to sequencing read depth (CPM; Counts per million). Expression levels of the T‐cell markers *CD3e*, *CD8b* and *CD4* were normalized as RPKM; reads per kilobase per million. Patients were divided into tertiles (1–3) of expression levels with the 1^st^ tertile being low expressors and 3^rd^ tertile being high expressors. Disease‐free survival was analyzed for each variable with a log‐rank test. (H) Venn diagram of overlapping samples in which CMV‐TCR, Flu‐TCR and EBV‐TCRs were detected in HLA‐A*02:01^+^ patients. (I) TCRβ reads as identified by MiXCR normalized to read depth (CPM; Counts per million reads) compared in CMV‐TCR^+^, Flu‐TCR^+^ and EBV‐TCR^+^ samples by Kruskal–Wallis‐test. (J) *CD8b* expression (RPKM; reads per kilobase per million) compared in CMV‐TCR^+^, Flu‐TCR^+^ and EBV‐TCR^+^ samples by Kruskal–Wallis‐test. Box plot with all samples shown. Ns = non significant. The box plot shows 25 percentile, median, 75 percentile and error bar shows the minimum and maximum values. All values are shown in graphs. [Colour figure can be viewed at wileyonlinelibrary.com]

A large number of genes were differentially expressed in tumors of HLA matched CMV‐TCR^+^ compared to CMV‐TCR^−^ patients with gene ontology terms relating to e.g. immune processes being upregulated (Fig. 6A and Fig. S5A,B, Table S8). There was no difference in immune cell composition in CMV‐TCR^+^ and CMV‐TCR^−^ tumors, except for a slight increase of CD8^+^ T‐cells in CMV‐TCR^+^ tumors (Fig. 6B). Substantial T‐cell infiltration is associated with lethal prostate cancer [28], but T‐cell infiltration levels were not associated with disease recurrence in CPC‐GENE (Fig. 6C–G). Thus, the association between CMV‐TCR and disease recurrence appeared independent of T‐cell numbers in tumors. Further supporting this, samples positive for Flu‐TCR and EBV‐TCR, for which there was no association with disease recurrence, had equal levels of T‐cell infiltration as samples positive for CMV‐TCR (Fig. 6H–J).

## 4 Discussion

In summary, we report that HLA‐A*02:01 and HLA‐A*11 are associated with a risk of disease recurrence after radical prostatectomy independent of Gleason score, s‐PSA and T‐stage in a cohort with low‐intermediate risk and a high‐risk prostate cancer cohort, respectively. This suggests that HLA drives the development of advanced prostate cancer by promoting metastases independent of cellular dedifferentiation or invasion. Further studies are required to validate an association with HLA‐A*02:01, HLA‐A*11 and prostate cancer prognosis. We envision that HLA‐type, determined in a blood test or prostate biopsy, can be incorporated into risk models to determine if a patient will develop aggressive prostate cancer or live with its indolent form.

HLA‐A*02 carriers, for example, have decreased risk of EBV^+^ Hodgins lymphoma [29] and an increased risk of ovarian cancer‐related mortality [30]. Thus, HLA‐A*02 can interact with tumor biology in multiple ways. An association between HLA‐A*11 and other cancer types is less apparent.

Cohorts that allow high throughput HLA typing and tumor TCR analysis with long‐term follow‐up are sparse, limiting our analysis to surrogate markers of prostate cancer relapse in a limited number of patients. As antiviral T‐cell responses are not well documented in HLA‐A*11 carriers, we were not able to interrogate this. In addition, we were unable to account for some other known prognostic factors such as positive surgical margins as this information was not available [31]. We can therefore not rule out positive surgical margins as a confounding factor.

Homozygous carriers of HLA‐A*02:01 were not more likely to relapse than heterozygous carriers, in contrast to *de novo* metastatic disease [4]. It is possible that additive effects of HLA‐A*02:01 are at play upon antiandrogen therapy. Androgens generally promote an immune‐suppressive environment, and androgen deprivation therapy, administered to patients with advanced prostate cancer, may alter the immune microenvironment in metastases [32].

Local T‐cell immunity to CMV, but not EBV or Influenza A, was associated with particularly poor disease‐free survival in HLA‐A*02:01 carriers. Our findings reveal prognostically relevant immune and viral interactions in prostate cancer that prompt further exploration and validation. CMV seropositivity is common and varies across the world [33]. Interestingly, CMV seropositive men, but not women, have an increased risk of dying from cancer [34]. Perhaps, this sex difference is due to increased prostate cancer mortality among men. Although CMV has been implicated in several tumor types, its association with prognosis and function has remained unclear. Of patients with glioblastoma, those who were CMV seropositive at diagnosis had poorer overall survival than CMV seronegative patients [35].

Local T‐cell immunity to CMV was associated with particularly poor disease‐free survival in HLA‐A*02:01 carriers. In contrast, antiviral T‐cell activation may potentiate antitumor responses in other tumor types [26, 36]. CMV‐directed T‐cell clones can expand drastically with age and are postulated to compromise immunity [37], potentially providing a hostile prostate cancer environment. Another possibility is that CMV may infect prostate cancer cells [38] and directly promote tumor progression. To this end, it can perhaps be possible to boost tumor immunity by activating potentially resting anti‐CMV T‐cells in prostate cancer. If so, CMV reactive T‐cells may not only be used as a prognostic biomarker but CMV itself could be a therapeutic target.

## 5 Conclusion

Variants of genes that are essential for the immune system (HLA‐A*02:01 and HLA‐A*11) were associated with prostate cancer progression after radical prostatectomy. Local detection of immunity to CMV was associated with particularly low disease recurrence‐free survival, suggesting that HLA‐type and viral immunity could predict prostate cancer progression.

## Conflict of interest

MZ, JF, CE and JM are consultants to 10× Genomics.

## Author contributions

JF had full access to all the data in the study and takes responsibility for the integrity of the data and accuracy of the data analysis. Study concept and design: JC, JF. Acquisition of data: JC, MZ, CE, KA, GM, CP, DN. Analysis and interpretation of data: JC, MZ, CE, HD, JM, JF. Drafting of the manuscript: JC, JF. Critical revision of the manuscript for important intellectual content: JC, MZ, CE, KA, GM, CP, DN, PB, HD, JM, JF. Statistical analysis: JC, MZ. Obtaining funding: JF. Supervision: PB, JF.

### Peer Review

The peer review history for this article is available at https://publons.com/publon/10.1002/1878‐0261.13273.

## Supporting information


**Fig. S1.** HLA‐typing validation.

**Fig. S2.** Additional information on the analysis of HLA and disease recurrence.

**Fig. S3.** Additional information on antiviral immunity in the prostate.

**Fig. S4.** EBV‐TCR and Flu‐TCR detection are not associated with prostate cancer disease recurrence.

**Fig. S5.** Additional information on antiviral immunity and prostate cancer disease recurrence.

**Table S1.** Description of CPC‐GENE and TCGA‐PRAD cohorts.

**Table S2.** Clinical characteristics of HLA‐A*02:01^−^ and HLA‐A*02:01^+^ prostate cancer patients in CPC‐GENE.

**Table S3.** CPC‐GENE alleles with over 5% in allele frequency.

**Table S4.** TCGA‐PRAD alleles with over 5% in allele frequency.

**Table S5.** Clinical characteristics of HLA‐A*11^−^ and HLA‐A*11^+^ prostate cancer patients in TCGA‐PRAD.

**Table S6.** Postmortem prostate donors and antiviral IgG analysis.

**Table S7.** Clinical characteristics of CMV‐TCR^−^ and CMV‐TCR^+^ HLA‐A*02:01^+^ prostate cancer patients.

**Table S8.** Differentially expressed genes in CMV‐TCR^−^ and CMV‐TCR^+^ samples in CPC‐GENE.

## Data Availability

CPC‐GENE and TCGA‐PRAD results including genetic information on individual research subject are not shared due to data sharing restrictions. Virscan results of each individual are shared in Table S6. Differentially expressed genes in CMV‐TCR^−^ and CMV TCR^+^ prostate tumors in CPC‐GENE are shared in Table S8.
