# Supplementary material for: Prostate cancer disease recurrence after radical prostatectomy is associated with HLA type and local cytomegalovirus immunity
Source: Mol Oncol. 2022 Aug 31;16(19):3452–64. doi: 10.1002/1878-0261.13273 (PMC9533687; doi:10.1002/1878-0261.13273)
Supplement: Supplementary file 1 — Fig. S1. HLA‐typing validation. [file MOL2-16-3452-s012.pdf]

A

row 1: germline WGS (HLA*LA) best guess
row 2: tumor RNA-seq (ArcasHLA) best guess

Patient	HLA-A		HLA-B		HLA-C		HLA-DPB1		HLA-DQA1		HLA-DQB1		HLA-DRB1	
1	32:01:01	01:01:01								05:01:01				
	32:01:01	32:01:01								05:05:01				
2											05:01:01			
											05:45			
3							04:02:01				02:01:01			
							105:01:01				02:02:01			
4														
5										05:01:01				
										05:05:01				
6		25:14								05:01:01				
		25:01:01								05:05:01				
7							39:01			01:01:01				
							40:01:01			01:01:02				
8														
9										01:01:01				
										01:04:02				
10				40:01:01						03:01:01				
				40:01:02						03:03:01				
11										03:01:01				
										03:02:01				
12										05:01:01				
										05:05:01				
13											05:01:01			
											05:45			

- (pink) Possible deletion, Loss of heterozygozity, in prostate cancer
- (green) HLA*LA best guess has 0% allele frequency for HLA-DQA*05:05:01, suggesting false genotypes for this allele.
- (blue) Difference within HLA-groups in HLA*LA and ArcasHLA genotypes
- (orange) Difference between HLA-groups (1-field) in HLA*LA and ArcasHLA genotypes
