# Supplementary figures and images for: Prostate cancer disease recurrence after radical prostatectomy is associated with HLA type and local cytomegalovirus immunity
Source: Mol Oncol. 2022 Aug 31;16(19):3452–64. doi: 10.1002/1878-0261.13273 (PMC9533687; doi:10.1002/1878-0261.13273)

A

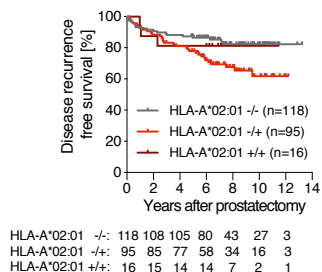

B

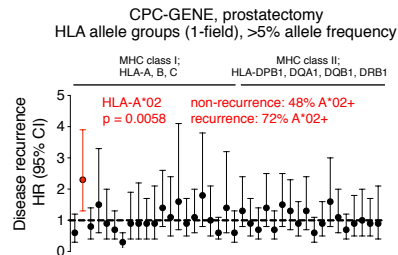

C

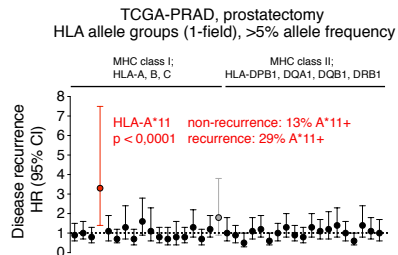

D

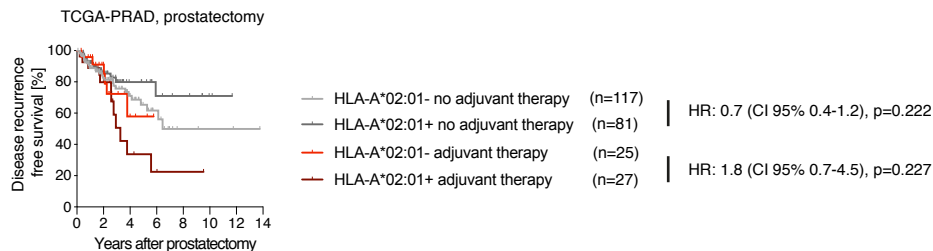

E

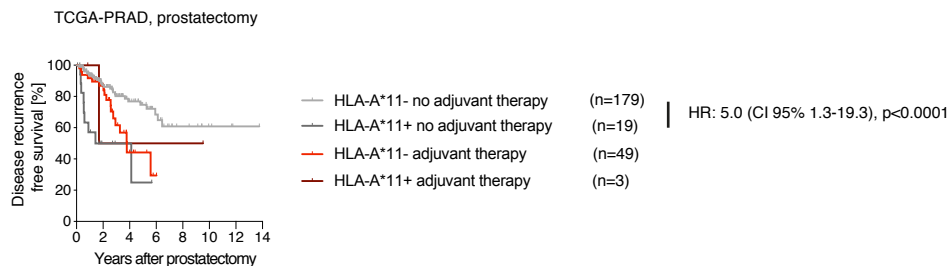

Supplement: Supplementary file 2 — Fig. S2. Additional information on the analysis of HLA and disease recurrence. [file MOL2-16-3452-s013.pdf]

**A**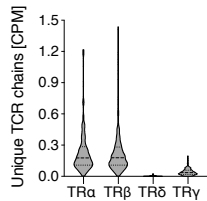**B**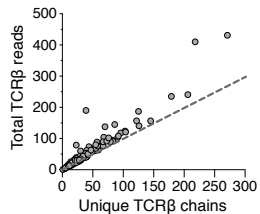**C**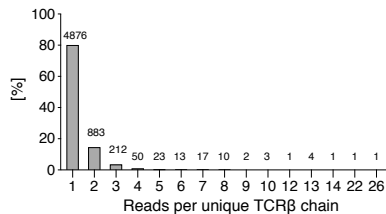**D**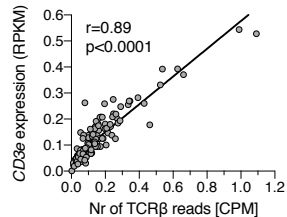**E**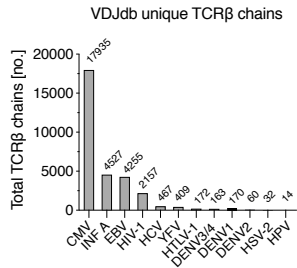**F**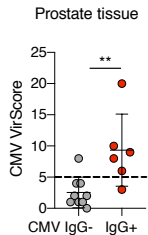**G**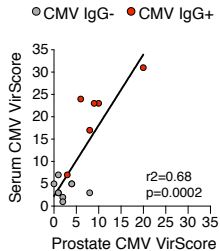

Supplement: Supplementary file 3 — Fig. S3. Additional information on antiviral immunity in the prostate. [file MOL2-16-3452-s002.pdf]

**A**

CMV-TCR+ compared to CMV-TCR-

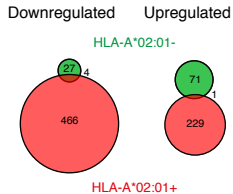**B**

HLA-A\*02:01+: Gene ontology: biological processes, top 10, down in CMV-TCR+

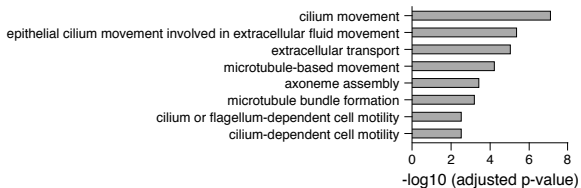

Supplement: Supplementary file 5 — Fig. S5. Additional information on antiviral immunity and prostate cancer disease recurrence. [file MOL2-16-3452-s008.pdf]
