# Supplementary material for: Prostate cancer disease recurrence after radical prostatectomy is associated with HLA type and local cytomegalovirus immunity
Source: Mol Oncol. 2022 Aug 31;16(19):3452–64. doi: 10.1002/1878-0261.13273 (PMC9533687; doi:10.1002/1878-0261.13273)
Supplement: Supplementary file 4 — Fig. S4. EBV‐TCR and Flu‐TCR detection are not associated with prostate cancer disease recurrence. [file MOL2-16-3452-s005.pdf]

A

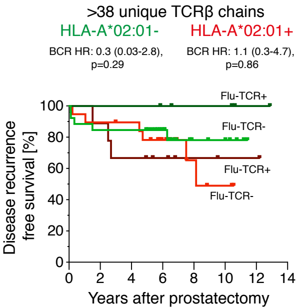

HLA-A*02:01- Flu-TCR-	26	23	23	16	7	6	1
HLA-A*02:01- Flu-TCR+	5	5	5	5	4	4	2
HLA-A*02:01+ Flu-TCR-	19	18	17	11	5	3	1
HLA-A*02:01+ Flu-TCR+	9	9	7	5	3	2	2

B

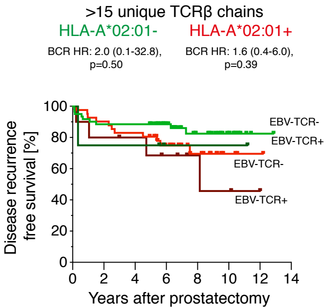

HLA-A*02:01- EBV-TCR-	61	55	55	39	21	13	2
HLA-A*02:01- EBV-TCR+	4	4	4	2	2	2	1
HLA-A*02:01+ EBV-TCR-	41	38	35	24	13	4	2
HLA-A*02:01+ EBV-TCR+	10	9	8	6	4	3	2
