# Supplementary material for: Prostate cancer disease recurrence after radical prostatectomy is associated with HLA type and local cytomegalovirus immunity
Source: Mol Oncol. 2022 Aug 31;16(19):3452–64. doi: 10.1002/1878-0261.13273 (PMC9533687; doi:10.1002/1878-0261.13273)
Supplement: Supplementary file 6 — Table S1. Description of CPC‐GENE and TCGA‐PRAD cohorts. [file MOL2-16-3452-s003.pdf]

**Supplementary Table 1**
**Description of CPC-GENE and TCGA-PRAD cohorts**

| | CPC-GENE (n=229) | TCGA-PRAD (n=441) |
|---|---|---|
| Age (years; median, min-max) | 62 (42-77) | 61 (43-77) |
| s-PSA (median, min-max) | 6,6 (1,7-39,5) | 7,5 (1,5-107,0)* |
| Gleason grade group (n (%)) | | |
| 1 | 16 (7) | 42 (10) |
| 2 | 135 (59) | 129 (29) |
| 3 | 61 (27) | 96 (22) |
| 4 | 12 (5) | 58 (13) |
| 5 | 5 (2) | 116 (26) |
| Missing data | 0 (0) | 0 (0) |
| T-stage (n (%))** | | |
| T1 | 123 (54) | 0 (0) |
| T2a | 59 (26) | 10 (2) |
| T2b | 42 (18) | 8 (2) |
| T2c | 5 (2) | 156 (35) |
| T3a | 0 (0) | 140 (32) |
| T3b | 0 (0) | 112 (25) |
| T4 | 0 (0) | 9 (2) |
| Missing data | 0 (0) | 6 (1) |
| N-stage (n (%)) | | |
| 0 | 229 (100) | 306 (69) |
| 1 | 0 (0) | 67 (15) |
| Missing data | 0 (0) | 68 (15) |
| Disease recurrence (n (%)) | | |
| 0 | 176 (77) | 356 (81) |
| 1 | 53 (23) | 85 (19) |
| Follow up time (years; median) | 6,8 | 2,1 |
| Follow up time to disease recurrence (years; median) | 2,5 | 1,4 |
| Follow up time to censured (years; median) | 7,7 | 2,3 |

* 14 TCGA-PRAD patients are missing pre-treatment s-PSA
** CPC-GENE: clinical T-stage; TCGA-PRAD: pathological T-stage
