# Supplementary material for: Prostate cancer disease recurrence after radical prostatectomy is associated with HLA type and local cytomegalovirus immunity
Source: Mol Oncol. 2022 Aug 31;16(19):3452–64. doi: 10.1002/1878-0261.13273 (PMC9533687; doi:10.1002/1878-0261.13273)
Supplement: Supplementary file 7 — Table S2. Clinical characteristics of HLA‐A*02:01− and HLA‐A*02:01+ prostate cancer patients in CPC‐GENE. [file MOL2-16-3452-s006.pdf]

Supplemental Table 2

Clinical characteristics of HLA-A*02:01- and HLA-A*02:01+ prostate cancer patients in CPC-GENE

	HLA-A*02:01- (n=188)	HLA-A*02:01+ (n=111)	
Age, years median (range)	62 (42-74)	62 (51-77)	Mann-Whitney p=0.89
s-PSA median (range)	6.5 (2.0-39.5)	6.7 (1.7-19.5)	Mann-Whitney p=0.56
Gleason grade group, n (%)			
1	9 (8)	7 (6)	Fisher's exact test
2	71 (60)	64 (58)	1-3 vs 4-5
3	28 (24)	33 (30)	p=0.79
4	7 (6)	5 (5)	
5	3 (3)	2 (2)	
T-stage (cT) n, (%)			
T1a-T1c	62 (53)	61 (55)	Fisher's exact test
T2a	33 (28)	26 (23)	T1 vs T2
T2b	19 (16)	23 (21)	p=0.62
T2c	4 (3)	1 (1)	
