# Supplementary material for: Prostate cancer disease recurrence after radical prostatectomy is associated with HLA type and local cytomegalovirus immunity
Source: Mol Oncol. 2022 Aug 31;16(19):3452–64. doi: 10.1002/1878-0261.13273 (PMC9533687; doi:10.1002/1878-0261.13273)
Supplement: Supplementary file 8 — Table S3. CPC‐GENE alleles with over 5% in allele frequency. [file MOL2-16-3452-s010.pdf]

Supplemental Table 3

List of Log-rank tests of HLA allele groups >5% allele frequency in CPC-GENE

HLA-allele group	Negative	Positive	HR recurrence	CI 95% low bound	CI 95% high bound
A*01	180	49	0,6	0,3	1,2
A*02	106	123	2,3	1,3	3,9
A*03	164	65	0,8	0,4	1,4
A*11	199	30	1,5	0,6	3,3
A*24	196	33	0,9	0,4	2
B*07	174	55	0,7	0,4	1,3
B*08	203	26	0,3	0,1	0,6
B*15	192	37	0,9	0,4	1,9
B*18	205	24	0,9	0,4	2,2
B*35	185	44	0,9	0,4	1,7
B*40	205	24	0,9	0,4	2,1
B*44	170	59	1,4	0,8	2,6
B*51	198	31	1,1	0,5	2,4
C*01	206	23	1,6	0,7	4,1
C*03	175	54	0,9	0,5	1,7
C*04	174	55	1,1	0,6	2
C*05	194	35	1,8	0,8	3,8
C*06	193	36	1	0,5	2,1
C*07	127	102	0,6	0,4	1,1
C*12	199	30	1,4	0,6	3,2
C*16	203	26	0,6	0,3	1,3
DQA1*01	68	161	1,3	0,8	2,4
DQA1*02	166	63	0,9	0,5	1,7
DQA1*03	167	62	0,7	0,4	1,4
DQA1*05	134	95	1,4	0,8	2,5
DQB1*02	154	75	0,7	0,4	1,3
DQB1*03	101	128	1,5	0,8	2,5
DQB1*05	154	75	1,3	0,7	2,3
DQB1*06	121	108	0,9	0,5	1,6
DRB1*01	174	55	1,3	0,7	2,4
DRB1*04	170	59	0,6	0,3	1,1
DRB1*07	165	64	0,9	0,5	1,6
DRB1*11	178	51	1,6	0,8	3
DRB1*13	170	59	1,1	0,6	2
DRB1*15	171	58	0,7	0,4	1,2
DPB1*02	177	52	0,9	0,5	1,8
DPB1*03	187	42	1	0,5	2
DPB1*04	63	166	0,9	0,5	1,7
DPB1*05	206	23	0,9	0,4	2,1
