# Supplementary material for: Prostate cancer disease recurrence after radical prostatectomy is associated with HLA type and local cytomegalovirus immunity
Source: Mol Oncol. 2022 Aug 31;16(19):3452–64. doi: 10.1002/1878-0261.13273 (PMC9533687; doi:10.1002/1878-0261.13273)
Supplement: Supplementary file 9 — Table S4. TCGA‐PRAD alleles with over 5% in allele frequency. [file MOL2-16-3452-s004.pdf]

Supplemental Table 4

List of Log-rank tests of HLA allele groups >5% allele frequency in TCGA-PRAD

HLA-allele group	Negative	Positive	HR recurrence	CI 95% low bound	CI 95% high bound
HLA-A*01	322	119	0,9	0,6	1,5
HLA-A*02	236	205	1	0,7	1,6
HLA-A*03	339	102	0,8	0,5	1,3
HLA-A*11	399	42	3,3	1,4	7,5
HLA-A*24	358	83	1,1	0,6	1,9
HLA-B*07	339	102	0,7	0,5	1,2
HLA-B*08	364	77	1,3	0,7	2,4
HLA-B*15	371	70	0,7	0,4	1,2
HLA-B*35	374	67	1,6	0,9	2,8
HLA-B*40	387	54	1,1	0,6	2,3
HLA-B*44	334	107	0,8	0,5	1,3
HLA-B*51	389	52	0,7	0,4	1,3
HLA-C*02	395	46	0,8	0,4	1,6
HLA-C*03	338	103	0,8	0,5	1,3
HLA-C*04	346	95	1,3	0,8	2,2
HLA-C*06	361	80	0,7	0,4	1,2
HLA-C*07	230	211	1,2	0,8	1,9
HLA-C*12	393	48	1,8	0,9	3,8
HLA-DPB1*01	376	65	1	0,6	1,8
HLA-DPB1*02	317	124	0,9	0,5	1,4
HLA-DPB1*03	369	72	0,5	0,3	1
HLA-DPB1*04	126	315	1,1	0,7	1,8
HLA-DQA1*01	142	298	1,2	0,8	1,9
HLA-DQA1*02	328	112	0,6	0,4	1
HLA-DQA1*03	306	134	1	0,6	1,5
HLA-DQA1*05	269	171	1,3	0,8	2
HLA-DQB1*02	275	166	0,9	0,6	1,4
HLA-DQB1*03	208	233	0,8	0,5	1,3
HLA-DQB1*05	293	148	1,3	0,8	2
HLA-DQB1*06	258	183	1,1	0,7	1,7
DRB1*01	355	86	1,2	0,7	2,1
DRB1*03	346	95	1,4	0,8	2,3
DRB1*04	323	118	1	0,6	1,6
DRB1*07	329	112	0,6	0,4	1
DRB1*11	353	88	1,4	0,8	2,4
DRB1*13	344	97	1,1	0,7	1,8
DRB1*15	338	103	1	0,6	1,7
