# Supplementary material for: Prostate cancer disease recurrence after radical prostatectomy is associated with HLA type and local cytomegalovirus immunity
Source: Mol Oncol. 2022 Aug 31;16(19):3452–64. doi: 10.1002/1878-0261.13273 (PMC9533687; doi:10.1002/1878-0261.13273)
Supplement: Supplementary file 10 — Table S5. Clinical characteristics of HLA‐A*11− and HLA‐A*11+ prostate cancer patients in TCGA‐PRAD. [file MOL2-16-3452-s007.pdf]

Supplemental Table 5

Clinical characteristics of HLA-A*11- and HLA-A*11+ prostate cancer patients in TCGA-PRAD

	HLA-A*11- (n=305)	HLA-A*11+ (n=31)	
Age, years median (range)	62 (43-77)	61 (47-73)	Mann-Whitney p=0.35
s-PSA median (range)	7.8 (1.5-107.0)	7.5 (3.6-33.7)	Mann-Whitney p=0.96
Gleason grade group, n (%)			
1	17 (6)	1 (3)	Fisher's exact test
2	82 (27)	10 (32)	1-3 vs 4-5
3	70 (23)	9 (29)	p=0.35
4	43 (14)	5 (16)	
5	93 (30)	6 (19)	
T-stage (cT) n, (%)			
T2a	3 (1)	1 (3)	Fisher's exact test
T2b	6 (2)	0 (0)	T2 vs T3-4
T2c	100 (33)	8 (26)	p=0.56
T3a	108 (35)	11 (35)	
T3b	81 (27	11 (35)	
T4	7 (2)	0 (0)	
N-stage n, (%)			Fisher's exact test
N1	57 (19)	5 (16)	p>0.99
