# Supplementary material for: Prostate cancer disease recurrence after radical prostatectomy is associated with HLA type and local cytomegalovirus immunity
Source: Mol Oncol. 2022 Aug 31;16(19):3452–64. doi: 10.1002/1878-0261.13273 (PMC9533687; doi:10.1002/1878-0261.13273)
Supplement: Supplementary file 12 — Table S7. Clinical characteristics of CMV‐TCR− and CMV‐TCR+ HLA‐A*02:01+ prostate cancer patients. [file MOL2-16-3452-s001.pdf]

Supplemental Table 7

Clinical characteristics of CMV-TCR- and CMV-TCR+ HLA-A*02:01+ prostate cancer patients

	HLA-A*02:01+, >23 TCRb chains		
	CMV-TCR- (n=28)	CMV-TCR+ (n=10)	
Age, years			Student's t-test
median (range)	61 (52-69)	63 (52-65)	p=0.98
s-PSA			Student's t-test
median (range)	5.8 (2.0-10.9)	7.7 (2.0-19.5)	p=0.07
Gleason grade group, n (%)			
1	2 (7)	0 (0)	Fisher's exact test
2	20 (71)	7 (70)	1-3 vs 4-5
3	6 (21)	2 (20)	p=0.26
4	0 (0)	1 (10)	
5	0 (0)	0 (0)	
T-stage (cT) n, (%)			
T1a-T1c	18 (64)	5 (50)	Fisher's exact test
T2a	8 (29)	3 (30)	T1 vs T2
T2b	2 (7)	2 (20)	p=0.47
T2c	0 (0)	0 (0)	
